# Comparison Between Contract–Relax Stretching and Antagonist Contract–Relax Stretching on Gastrocnemius Medialis Passive Properties

**DOI:** 10.3389/fphys.2021.764792

**Published:** 2022-02-04

**Authors:** Taizan Fukaya, Andreas Konrad, Shigeru Sato, Ryosuke Kiyono, Kaoru Yahata, Koki Yasaka, Remi Onuma, Riku Yoshida, Masatoshi Nakamura

**Affiliations:** ^1^Institute for Human Movement and Medical Sciences, Niigata University of Health and Welfare, Niigata, Japan; ^2^Department of Rehabilitation, Kyoto Kujo Hospital, Kyoto, Japan; ^3^Institute of Human Movement Science, Sport and Health, Graz University, Graz, Austria; ^4^Department of Physical Therapy, Niigata University of Health and Welfare, Niigata, Japan

**Keywords:** contract-relax stretching, antagonist contract-relax stretching, range of motion, stretch tolerance, shear elastic modulus

## Abstract

Antagonist contract-relax stretching and contract-relax stretching is commonly used in sports practice and rehabilitation settings. To date, no study has compared these modalities regarding muscle stiffness and stretch tolerance. This study aimed to investigate the effects of contract-relax and antagonist contract-relax stretching on dorsiflexion range of motion (ROM), stretch tolerance, and shear elastic modulus. Forty healthy participants (24 men and 16 women) took part in the study. Participants were randomly assigned to perform either contract-relax stretching or antagonist contract-relax stretching for 2 min. Outcomes were assessed on ROM, stretch tolerance, and shear elastic modulus before and after stretching. The ROM and stretch tolerance significantly increased after both contract-relax stretching (+ 5.4 ± 5.8°, *p* < 0.05; + 3.5 ± 8.0 Nm, *p* < 0.05) and antagonist contract-relax stretching (+ 6.1 ± 4.9°, *p* < 0.05; + 4.2 ± 6.4 Nm, *p* < 0.05); however, no significant difference was found between the two groups. Alternatively, the shear elastic modulus significantly decreased after both contract-relax (–31.1 ± 22.6 kPa, *p* < 0.05) and antagonist contract-relax stretching (–11.1 ± 22.3 kPa, *p* < 0.05); however, contract-relax stretching (–41.9 ± 19.6%) was more effective than antagonist contract-relax stretching (–12.5 ± 61.6%). The results of this study suggest that contract-relax stretching instead of antagonist contract-relax stretching should be conducted to decrease muscle stiffness. However, either contract-relax or antagonist contract-relax stretching can increase ROM.

## Introduction

Range of motion (ROM), which is the ability to move a joint and ease muscle stiffness, is essential in sports performance and activities of daily living (Mulholland and Wyss, 2001; Hemmerich et al., 2006), and it might influence the risk of muscle strain injury (Witvrouw et al., 2003). In sports and clinical settings, static stretching (SS) is a common and easy technique to increase ROM, which is involved in changing the viscoelastic properties of the muscle-tendon unit (i.e., decreasing muscle stiffness) (Morse et al., 2008) and increasing an individual’s capacity to tolerate loading before stretch termination (i.e., increased stretch tolerance) (Magnusson et al., 1996).

Previous studies have shown that ROM significantly increased after acute and chronic interventions using proprioceptive neuromuscular facilitation (PNF) stretching compared with using SS (Funk et al., 2003; Hindle et al., 2012). A common method of PNF stretching is the contract-relax (CR) technique (Sharman et al., 2006), which includes an SS phase for a prescribed duration, followed immediately by a maximal isometric contraction in a fully stretched position. Kay et al. (2015) reported that CR stretching was more effective in increasing ROM and decreasing passive muscle-tendon stiffness than SS. Alternatively, Ferber et al. (2002) reported that antagonist CR (ACR) stretching, which is a combination of SS and voluntary contraction of the antagonist muscle group in a stretched position, that is, “active stretching,” was more effective in changing ROM than CR stretching for older participants. However, to the best of our knowledge, no study has investigated the effect of ACR stretching on muscle stiffness and stretch tolerance in younger participants. Furthermore, no study has compared ACR stretching with CR stretching regarding these outcomes.

Recently, the shear elastic modulus was measured as the quantitative outcome of muscle stiffness using shear wave elastography (SWE) (Lacourpaille et al., 2012; Inami and Kawakami, 2016). A previous study reported a positive correlation between the decrease in shear elastic modulus of the medial gastrocnemius (MG) and a decrease in muscle stiffness of MG (Nakamura et al., 2014). Additionally, Watsford et al. (2010) measured the hamstring muscle stiffness using a free oscillation technique and showed that excessive hamstring muscle stiffness causes hamstring strain injuries (Watsford et al., 2010). Therefore, it is important to decrease the shear elastic modulus to prevent injury. Studies have shown that the shear elastic modulus acutely and chronically decreased after SS (Ichihashi et al., 2016; Nakamura et al., 2017). Additionally, the shear elastic modulus decreased after both active stretching and SS; however, no significant difference was observed between active stretching and SS (Nakao et al., 2018). Although previous studies have investigated the effect of CR stretching on the shear elastic modulus (Reiner M. et al., 2021) or muscle stiffness (Nakamura et al., 2015, 2021), no study has compared the effect of CR and ACR stretching on the shear elastic modulus. Thus, it is important to investigate which of CR and ACR stretching is the better method to increase ROM. Additionally, we would like to clarify the difference in the change in stretch tolerance and shear elastic modulus, which is likely associated with the increasing ROM between both techniques.

Therefore, this study aimed to examine and compare the effect of CR and ACR stretching on ROM, stretch tolerance, and shear elastic modulus. Hypothetically, ACR stretching is more effective in changing ROM, stretch tolerance, and shear elastic modulus than CR stretching because the previous study has shown that changes in ROM were greater after ACR stretching than after CR stretching (Ferber et al., 2002).

## Materials and Methods

### Experimental Design

Participants were randomly assigned to the CR and ACR stretching groups. The characteristics of the participants are shown in Table 1, and no significant differences were found in each characteristic value between the CR and ACR stretching groups. CR and ACR stretching was performed for 2 min (30 s × 4 sets, with no rest) for plantar flexor muscles. To examine the effects of CR and ACR stretching, dorsiflexion (DF) ROM, passive torque at DF ROM, and shear elastic modulus of the MG muscle in the dominant leg (preferred to kick a ball) were measured before (PRE) and after (POST) stretching.

**TABLE 1:** Physical characteristics of the subjects.

Characteristics	CR stretching groups	ACR stretching groups	*P*-value
Participants (male, female)	*N* = 20 (12, 8)	*N* = 20 (12, 8)	1
Age (years)	21.7 ± 2.5	21.7 ± 2.0	1
Height (cm)	166.6 ± 8.7	165.9 ± 7.7	0.8
Body mass (kg)	59.7 ± 10.3	60.3 ± 8.6	0.8
Body mass index (kg/m^2^)	21.4 ± 2.1	21.9 ± 2.3	0.5

*CR, contract-relax; ACR, agonist contract-relax.*

### Participants

Forty healthy young adults (24 men and 16 women) took part in the study (mean age, 21.7 ± 2.3 years; mean height, 166.2 ± 8.1 cm; mean body mass, 60.0 ± 9.4 kg). Previous studies have not investigated separately the effect of SS on men and women (Ryan et al., 2009; Kay et al., 2015). Therefore, this study did not separate men and women. Those who had a history of surgery on their back or lower body, lower-extremity contracture, and neurological disorders and those who took hormone- or muscle-affecting drugs were excluded from the study.

Written informed consent was obtained from all participants. Additionally, this study was approved by the ethics committee of our institution (approval no. 17677).

After calculating the sample size required for a split-plot analysis of variance (ANOVA) (effect size = 0.40 [large], α error = 0.05, and power = 0.80) using G* power 3.1 software (Heinrich Heine University, Düsseldorf, Germany) based on a previous study (Ferber et al., 2002), the minimum required number of participants in each group was 14.

### Measurement of Dorsiflexion Range of Motion and Passive Torque at Dorsiflexion Range of Motion

**FIGURE 1:**
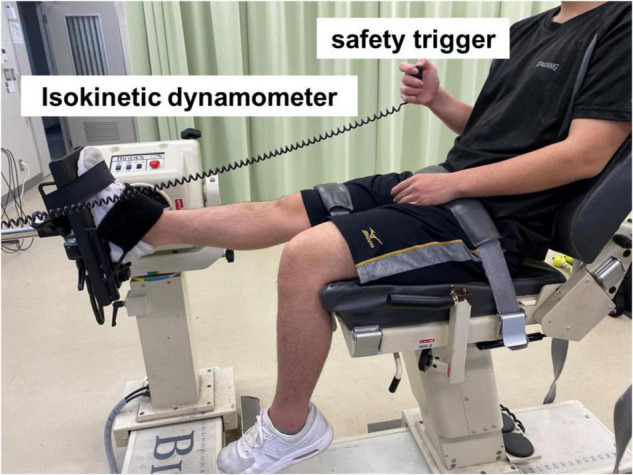
Experimental set-up for measuring the dorsiflexion range of motion and passive torque.

The participants were seated in an isokinetic dynamometer (System 3.0; Biodex Medical Systems, Inc., Shirley, NY, United States) chair at 0° knee angle (i.e., anatomical position) and 70° hip flexion to prevent tension at the back of the knee, with adjustable belts over the trunk and pelvis and ankle fixed to a footplate (Figure 1; Nakamura et al., 2020). Then, they moved the footplate of the dynamometer at a speed of 5° per s, starting from the ankle at 0° to the maximum DF angle without feeling pain until stopping the dynamometer by activating a safety trigger (Morse et al., 2008). DF ROM and passive torque were calculated from the torque–angle curve using the dynamometer (Nakamura et al., 2020; Sato et al., 2020). DF ROM (°) was defined as the maximum DF angle at the torque–angle curve. Passive torque at DF ROM (Nm) was calculated from the passive torque at the point of the DF angle at the torque–angle curve and was defined as the stretch tolerance (Mizuno et al., 2013). Two trials were conducted. The largest DF angle and the passive torque at the largest DF angle were used for further analysis (Blazevich et al., 2014).

### Measurement of Shear Elastic Modulus of the Medial Gastrocnemius Muscle

Ultrasonic SWE (Aplio 500, Toshiba Medical Systems, Tochigi, Japan) was used with a 5–14 MHz linear probe to measure the shear elastic modulus of the MG. Participants were positioned similar to the position during DF ROM measurement. The shear elastic modulus of the MG at 10° DF was measured at 30% of the lower leg length from the popliteal crease to the lateral malleolus near the point at which the maximal cross-sectional area in the lower leg is located (Nakamura et al., 2014, 2019). Ultrasound image measurements were conducted twice using long-axis images of the MG. The analysis of the shear wave speed in ultrasound images was conducted using an MSI Analyzer (version 5.0; Rehabilitation Science Research Institute, Japan). The measurement of the shear wave speed (Vs) was set as the region of interest in the area as large as possible in the MG, and the average value of the shear wave speed inside this region was obtained. The shear elastic modulus was calculated as μ (kPa) = ρVs^2^, where ρ is the muscle mass density (1,000 kg/m^3^). The average value of the shear elastic modulus obtained from two ultrasound images was used for analysis.

### Contract-Relax and Antagonist Contract-Relax Stretching Interventions and Measurement of the Stretching Angle

CR and ACR stretching were performed for the plantar flexor muscles in a position similar to that during DF ROM measurement. CR and ACR stretching were performed based on a previous study (Nakamura et al., 2015). Initially, the ankle was passively dorsiflexed at a speed of 5° per second from 0° angle to the maximal angle and was held for 15 s without feeling pain. In the CR stretching groups, participants were instructed to perform a maximal voluntary isometric contraction of the plantar flexors for 5 s in the stretching position. In the ACR stretching groups, participants performed a maximal voluntary isometric contraction of the dorsiflexors for 5 s in the stretching position. After this contraction, the ankle was held at the angle for an additional 10 s. After 30 s of CR and ACR stretching, the ankle was returned to 0° angle and was immediately moved to a new angle without rest to perform the next stretching. CR or ACR stretching was repeated four times for a total time of 2 min. The angle during stretching among sets of stretching was measured by using the isokinetic dynamometer.

### Measurement Reliability

The test-retest reliabilities of the DF ROM, passive torque at the DF ROM, and shear elastic modulus of the MG in seven healthy young adults (three men and four women) were investigated on different days. The calculated intraclass correlation coefficients (1,1) for the DF ROM, passive torque at DF ROM, and shear elastic modulus were 0.91 (95% confidence interval (CI) 0.63–0.98), 0.85 (95% CI 0.42–0.97), and 0.82 (95% CI 0.32–0.97), respectively, which indicates high reliability for all outcome measures (Landis and Koch, 1977).

### Statistical Analysis

Differences in all variables at PRE stretching between CR and ACR stretching were investigated using an unpaired *t*-test. To analyze the interaction effect of the DF ROM, passive torque at DF ROM, and shear elastic modulus of the MG, we conducted a split-plot ANOVA [time (PRE vs. POST) and conditions (CR vs. ACR stretching)]. Furthermore, if the interaction effect or main effect was significant, we determined the significant differences between PRE and POST using a paired *t*-test in each protocol as a *post hoc* test. Additionally, the Mann–Whitney *U*-test was used to evaluate the relative changes from PRE to POST to compare CR and ACR stretching because the relative changes were not normally distributed in the Shapiro–Wilk test. To evaluate the stretching intensity between CR and ACR stretching, we conducted a split-plot ANOVA [sets (1 vs. 2 vs. 3 vs. 4 sets) and conditions (CR vs. ACR stretching)] for the angle during CR and ACR stretching. Furthermore, if the interaction effect or main effect was significant, we conducted a Bonferroni test as a *post hoc* test. The effect size (ES) was calculated when the outcome was applied to the parametric tests using the F ratio and sample size. By contrast, using *z*-value and sample size, ES was calculated when the outcome was applied to non-parametric tests. The ES classification (Cohen’s d) was as follows: *r* < 0.1, trivial; 0.1–0.3, small; 0.3–0.5, moderate; and > 0.5, large (Cohen, 1988). All statistical analyses were conducted using R (version 2.8.1; The R Foundation, Vienna, Austria), and significance was set at *p* < 0.05. All data were presented as mean ± standard deviation.

## Results

### Dorsiflexion Range of Motion

**FIGURE 2:**
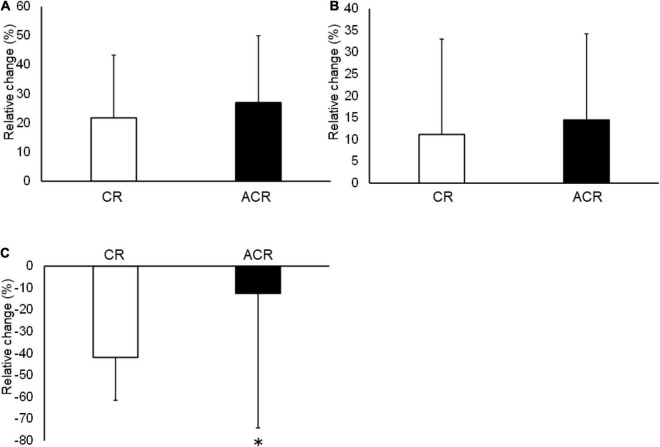
The relative changes in dorsiflexion (DF) range of motion (ROM) **(A)**, passive torque at DF ROM **(B)**, and shear elastic modulus **(C)** after contract-relax (CR) stretching and antagonist contract-relax (ACR) stretching. The relative changes in DF ROM and passive torque at DF ROM were no significant differences between CR and ACR stretching. The relative change in shear elastic modulus after CR stretching was significantly higher than ACR stretching (*p* < 0.01). *p < 0.05 compared with CR stretching.

No significant difference was found between CR and ACR stretching for DF ROM at PRE stretching (*p* = 0.52). The split-plot ANOVA did not indicate a significant interaction for DF ROM (*F* = 0.15, ES = 0.06; *p* = 0.71); however, a significant main effect for time was found (*F* = 45.52, ES = 0.74; *p* < 0.05). The *post hoc* test revealed a significant increase in DF ROM after both CR and ACR stretching similarly (Table 2). The relative change in DF ROM was no significant difference between CR and ACR stretching (Figure 2A).

**TABLE 2:** Acute effects of contract-relax (CR) stretching and antagonist contract-relax (ACR) stretching on dorsiflexion (DF) range of motion (ROM), passive torque at DF ROM, and shear elastic modulus at before (PRE), and after (POST) stretching intervention.

	DF ROM (°)		Passive torque at DF ROM (Nm)		Shear elastic modulus (kPa)	
	CR groups	ACR groups	CR groups	ACR groups	CR groups	ACR groups
PRE	25.7 ± 7.5	24.2 ± 6.5	29.7 ± 9.4	27.6 ± 10.2	23.4 ± 10.8	19.5 ± 5.4
POST	31.1 ± 10.0*	30.3 ± 8.4*	33.2 ± 14.1*	31.9 ± 14.2*	13.1 ± 6.9*	15.8 ± 11.7*

Data are presented as mean ± standard deviation. *p < 0.05 compared with PRE.

### Passive Torque at Dorsiflexion Range of Motion (Stretch Tolerance)

No significant difference was noted between CR and ACR stretching for the passive torque at DF ROM at PRE stretching (*p* = 0.51). The split-plot ANOVA did not indicate a significant interaction for the passive torque at DF ROM (*F* = 0.11, ES = 0.05; *p* = 0.74); however, a significant main effect for time was found (*F* = 11.22, ES = 0.48; *p* < 0.05). The *post hoc* test revealed a significant increase in the passive torque at DF ROM after both CR and ACR stretching (Table 2). The relative change in passive torque at DF ROM was no significant difference between CR and ACR stretching (Figure 2B).

### Shear Elastic Modulus of the Medial Gastrocnemius Muscle

No significant difference was found between CR and ACR stretching for the shear elastic modulus at PRE stretching (*p* = 0.29). The split-plot ANOVA indicated a significant interaction for the shear elastic modulus of the MG (*F* = 7.94, ES = 0.42; *p* < 0.05). The *post hoc* test revealed a significant decrease in the shear elastic modulus after both CR and ACR stretching (*p* < 0.05) (Table 2). Furthermore, the relative change in the shear elastic modulus after CR stretching (–41.9 ± 19.6%) was significantly higher than that after ACR stretching (–12.50 ± 61.6%, *p* < 0.01, ES = 0.48) (Figure 2C).

### Stretching Angle

The stretching angle data in both CR and ACR stretching are shown in Table 3. The split-plot ANOVA did not indicate a significant interaction for the stretching angle (*F* = 0.72, ES = 0.14; *p* = 0.46); however, a significant main effect for time was found (*F* = 73.67, ES = 0.81; *p* < 0.05). The stretching angle increased over sessions in both CR and ACR stretching, and the stretching angle increased significantly from the first set to the second, third, and fourth sets (*p* < 0.05). Also, the stretching angle increased significantly from the second set to the third and fourth sets in both CR and ACR stretching conditions.

**TABLE 3:** Stretching angle during contract-relax (CR) stretching and antagonist contract-relax (ACR) stretching.

Number of sets	First set	Second set	Third set	Fourth set
**Stretching angle (°)**				
CR group	24.5 ± 8.1	28.4 ± 8.1*	29.5 ± 8.2*	30.6 ± 9.3*^†^
ACR group	24.2 ± 5.8	28.5 ± 7.2*	30.2 ± 8.0*^†^	31.6 ± 8.2*^†‡^

*Data are presented as mean ± standard deviation.*

*p < 0.05 compared with first set, ^†^p < 0.05 compared with second set, ^‡^p < 0.05 compared with third set.

## Discussion

This study examined and compared the effect of CR and ACR stretching on ROM, stretch tolerance, and shear elastic modulus. Our results revealed that DF ROM and stretch tolerance were significantly increased after stretching; however, no significant difference was observed between CR and ACR stretching. Alternatively, the shear elastic modulus of the MG has significantly decreased after both CR and ACR stretching. The relative decrease change in the shear elastic modulus after CR stretching was higher than that after ACR stretching. To our knowledge, this is the first to show that the effects of CR and ACR stretching on ROM and stretch tolerance have no significant difference. However, CR stretching was more effective in changing the shear elastic modulus than ACR stretching.

This study showed that DF ROM significantly increased after CR and ACR stretching. The increase in DF ROM after CR stretching was 21.7 ± 21.6%, which was similar to the results of a previous study (17.2 ± 4.4%) performing CR stretching for 2 min (Nakamura et al., 2015). Furthermore, a study reported that ROM increased after ACR stretching for 80 s (Ferber et al., 2002), which was slightly shorter than that in the present study. A study has suggested that longer-duration stretching is more effective in increasing ROM (Matsuo et al., 2013). Therefore, we suggest that 120 s of ACR stretching is sufficient to increase DF ROM. However, contrary to our hypothesis based on a previous study (Ferber et al., 2002), the increase in DF ROM was not significantly different between CR and ACR stretching. Studies have reported that the increase in ROM after stretching depends on the intensity of stretching (Freitas et al., 2015a; Kataura et al., 2017; Takeuchi and Nakamura, 2020). In the present study, the angle during stretching was measured as the index of stretching intensity. The results showed that the stretching angle was not significantly different between CR and ACR stretching; as a result, no differences were found in the change in DF ROM between the CR and ACR stretching groups. Moreover, Ferber performed the stretching manually (Ferber et al., 2002), while in the present study, stretching was performed by using an isokinetic dynamometer. Therefore, the result was different when compared with those of the previous study because the stretching methods were different.

Our results showed that the passive torque at DF ROM significantly increased after both CR and ACR stretching. The passive torque at DF ROM was measured as an index of stretch tolerance, which is the capacity to tolerate loading before stretch termination (Halbertsma and Goeken, 1994; Nakamura et al., 2015). In this study, the relative increase change in the passive torque at DF ROM after CR stretching was 11.7%. Nakamura et al. (2015) reported that the relative increase change in stretch tolerance after hold–relax stretching was 14.5%, similar to the results of the present study. Moreover, the relative increase change in stretch tolerance after ACR stretching was 15.3%, and no significant difference was found between CR and ACR stretching. Nakamura et al. (2015) reported that the relative increase change in the passive torque at DF ROM after SS was 4.7%, which was lower than that after CR and ACR stretching in the present study. However, the mechanism of increasing stretch tolerance after stretching or contraction is still unclear. Studies have suggested that increases in stretch tolerance are caused by reduced perceptions of pain and discomfort, accompanied by a change in neural and psychological factors after stretching (Folpp et al., 2006; Law et al., 2009). In a future study, examining the effects of a target or antagonist muscle contraction at the stretching position on stretch tolerance is warranted.

This study found that the shear elastic modulus of the MG significantly decreased after both CR and ACR stretching. A study reported that the shear elastic modulus significantly decreased after 120 s of SS (Nakamura et al., 2014). The same duration was used in the present study. However, no study has compared the effect of CR and ACR stretching on the shear elastic modulus. The relative decrease change in the shear elastic modulus of the MG after ACR stretching was significantly lower than that after CR stretching. Nakao et al. (2018) reported that when comparing the effects of active stretching with those of passive stretching on the shear elastic modulus of the hamstrings, no significant difference was found between active stretching and SS intervention. ACR stretching is a combination of passive and active stretching, but ACR stretching could not obtain the combination effects on the shear elastic modulus. Furthermore, Nakamura et al. (2014) reported that the relative decrease change in the shear elastic modulus of the MG after SS intervention for 2 min was approximately –18% at 30° DF or –10% at 0° DF. The present study showed that the relative decrease change in the shear elastic modulus after ACR stretching was –19%, and this result was similar to that in the previous study (Nakamura et al., 2014), which was smaller than that after CR stretching (–41.9%). Kay et al. (2015) reported that passive joint stiffness after CR stretching greatly decreased compared with that after SS intervention. Chino and Takahashi (2015) reported that a higher positive correlation (*r* = 0.80) was found between the shear elastic modulus of the MG by SWE and passive joint stiffness. Therefore, when we considered the results of previous and present studies, we suggested that CR stretching was more effective in decreasing muscle stiffness than ACR stretching. However, the detailed mechanism of decreasing the shear elastic modulus after CR and ACR stretching is unknown. A previous study revealed that active stretching has a neurophysiological effect that causes reciprocal suppression (i.e., Ia suppression) by the contraction of antagonist muscles (Herda et al., 2013). Thus, ACR stretching could generate muscle tone relaxation due to reciprocal suppression. By contrast, a previous study reported that the stretching effect generated by CR stretching was a result of autogenic inhibition (Hindle et al., 2012). Regarding autogenic inhibition, neuromuscular inhibition was thought to occur as the tendon loading during the contraction phase of CR stimulated type Ib muscle afferent output from the Golgi tendon organs, stimulating spinal inhibitory synapses and hyperpolarizing the dendritic ends of spinal α-motoneurons of the stretched muscle (Hindle et al., 2012). Therefore, autogenic inhibition (CR stretching) may be more effective in decreasing the shear elastic modulus than reciprocal suppression (ACR stretching). However, electromyography was not measured in this study, and future studies should investigate the effect of neurological properties after CR and ACR stretching.

A study reported that compared with controls, the shear modulus in patients with stroke increased in the gastrocnemius muscle when the knee was extended (Le Sant et al., 2019). Additionally, the shear modulus increased after performing eccentric exercises on several muscles (Pournot et al., 2016; Lacourpaille et al., 2017). Therefore, decreasing shear elastic modulus is important in athletic and clinical fields, and CR stretching should be performed more than ACR stretching based on the results of the present study. In this study, stretching was conducted using an isokinetic dynamometer, which is not a clinical method. However, Kay et al. (2020) reported no significant difference between field-based CR stretching without a dynamometer and CR stretching using a dynamometer in changing ROM and passive joint stiffness. Therefore, the results of this study could be applied in clinical practice.

This study has some limitations. First, the intraclass correlation coefficients of the CI for the shear elastic modulus are wide. However, the CIs of previous studies were similar to the results of this study (Nakamura et al., 2014; Fukaya et al., 2020). Therefore, future studies should use foils on the skin and previous B-mode images of the participants to better reproduce the US-probe placement and, hence, to optimize the reliability (Reiner M. et al., 2021; Reiner M. M. et al., 2021). Second, the stretching intensity was different among participants, as DF ROM was defined as the maximum tolerable ROM without pain. Freitas et al. (2015b) examined ROM using verbal and visual analog scales to evaluate the stretching intensity; these scales should be used to define stretching intensity based on ROM. Therefore, we should unify the stretching intensity between participants by using a verbal or visual analog scale.

## Conclusion

The results of this study revealed that DF ROM and stretch tolerance significantly increased after both CR and ACR stretching; however, no difference was found between the CR and ACR stretching groups. The shear elastic modulus of the MG significantly decreased after both CR and ACR stretching, and the relative decrease change in the shear elastic modulus of the MG after CR stretching was greater than that after ACR stretching. Therefore, either CR or ACR stretching may be performed to increase ROM; however, CR stretching is preferred to decrease muscle stiffness.

## Data Availability Statement

The raw data supporting the conclusions of this article will be made available by the authors, without undue reservation.

## Ethics Statement

The present study was approved by the ethics committee of our institution (approval number: 17677). The patients/participants provided their written informed consent to participate in this study.

## Author Contributions

TF, AK, RK, SS, KaY, KoY, RO, RY, and MN: conceptualization, investigation, and methodology. TF, RK, SS, KaY, KoY, RO, RY, and MN: investigation and methodology. TF and MN: data curation. AK and MN: funding acquisition, writing—review and editing, and supervision. MN: project administration. TF: visualization, formal analysis, and writing—original draft. All authors have read and agreed to the published version of the manuscript.

## Conflict of Interest

The authors declare that the research was conducted in the absence of any commercial or financial relationships that could be construed as a potential conflict of interest.

## Publisher’s Note

All claims expressed in this article are solely those of the authors and do not necessarily represent those of their affiliated organizations, or those of the publisher, the editors and the reviewers. Any product that may be evaluated in this article, or claim that may be made by its manufacturer, is not guaranteed or endorsed by the publisher.
